# MUC5AC and a Glycosylated Variant of MUC5B Alter Mucin Composition in Children With Acute Asthma

**DOI:** 10.1016/j.chest.2017.07.001

**Published:** 2017-07-14

**Authors:** Kathryn G. Welsh, Karine Rousseau, Gemma Fisher, Luke R. Bonser, Peter Bradding, Chris E. Brightling, David J. Thornton, Erol A. Gaillard

**Affiliations:** a Institute for Lung Health, National Institute for Health Research Leicester Biomedical Research Centre, and Department of Infection Immunity and Inflammation, University of Leicester, and University Hospitals Leicester, Children's Hospital, Leicester, England; b Wellcome Centre for Cell-Matrix Research, Faculty of Biology, Medicine and Health, Manchester Academic Health Science Centre, University of Manchester, Manchester, England; c Institute for Lung Health, National Institute for Health Research Leicester Respiratory Biomedical Research Unit, and Department of Infection Immunity and Inflammation, University of Leicester, and University Hospitals Leicester, Glenfield Hospital, Leicester, England

**Keywords:** airway obstruction, exacerbation, mucus, pediatric, sputum plug

## Abstract

**Background:**

Diffuse airway mucus obstruction is an important feature of severe and fatal asthma. MUC5AC and MUC5B are the principal gel-forming mucins found in airway mucus. The mucin composition of airway mucus likely affects its functional properties.

**Methods:**

We quantified the principal airway mucins MUC5AC and MUC5B in the sputum of age-matched children with acute and stable asthma and healthy control subjects by using Western blotting.

**Results:**

Sputum samples from 38 children (13 with acute asthma, 15 with stable asthma, 10 control subjects) were obtained. Sputum MUC5AC concentrations were 7.6 μg/mL in control subjects, 22.4 μg/mL in those with stable asthma (*P* = .17), and 44.7 μg/mL in those with acute asthma (*P* < .05). MUC5B concentrations showed less variation, with 238.5, 208.4 and 165.9 μg/mL in control subjects, those with stable asthma, and those with acute asthma, respectively. The greater MUC5AC concentration in those with acute asthma resulted in a significantly altered MUC5B:MUC5AC ratio between control subjects and those with acute asthma (*P* < .05). Significant differences in MUC5B glycoforms were present between the groups, with the low-charge-only glycoform being found uniquely in those with acute asthma.

**Conclusions:**

Increased MUC5AC and the presence of a low-charge-only MUC5B glycoform significantly altered mucin composition in children with acute asthma. These changes may be important contributory factors to the airway mucus obstruction observed during acute asthma.

Asthma exacerbations are common acute medical emergencies carrying significant morbidity and mortality.^1^ Exacerbations are triggered by a variety of factors, including viruses, aeroallergens, and exercise, and result in airway narrowing by a combination of airway wall edema, smooth muscle contraction, and airway mucus obstruction.^2^, ^3^ The latter is a major contributor to fatal asthma in children^4^ and adults.^5^, ^6^ Despite the consistent findings of airway remodeling with goblet cell hyperplasia^3^, ^7^ and submucosal gland hypertrophy^5^, ^8^ in both children and adults with asthma,^9^, ^10^ few studies have examined airway mucin composition in asthma,^7^, ^9^, ^10^, ^11^, ^12^, ^13^, ^14^, ^15^ and, to our knowledge, none have done so in children.

Mucins are large glycoproteins that provide the structural framework of mucus that protects the airway surface and that are important components of host innate defense.^7^, ^16^, ^17^, ^18^ MUC5AC is highly expressed in goblet cells, whereas MUC5B is largely secreted from airway submucosal glands.^19^ MUC5B exists as different glycosylated variants that can be distinguished on the basis of their electrophoretic mobility: a high-charge, fast-migrating glycoform and a low-charge, slow-migrating glycoform.^20^ Knowledge of the mucin composition of airway mucus is important because its physical properties depend largely on the high-molecular-weight, heavily glycosylated, polymeric gel-forming mucins MUC5AC and MUC5B.^20^, ^21^, ^22^ Changes in the mucin composition of mucus likely affect mucus rheologic properties,^23^ clearance,^24^ and plug formation.^11^

Previous research has shown that mucins are more abundant in the sputum of adults with asthma than in healthy control subjects,^9^ with MUC5AC predominating.^13^, ^20^ Mucin degradation in acute asthma has been shown to be inhibited in a protease-dependent manner,^15^ and tethering of MUC5AC to goblet cells impairs mucociliary clearance in asthma.^14^ Furthermore, the low-charge glycoform of MUC5B^11^, ^25^ is localized within sputum plugs obtained after fatal asthma.^11^

Despite an increasing appreciation of the importance of airway mucin composition in asthma,^4^, ^5^, ^6^ there have been no studies, to our knowledge, in children. Our principal aim was to compare and quantify sputum mucin composition in children with acute asthma and those with stable asthma with that of healthy control subjects.

## Materials and Methods

### Study Participants

Children aged 5 to 16 years seeking care for asthma were eligible to participate. Two groups with asthma were recruited. First, children with acute asthma diagnosed by the attending medical team in the ED were recruited; second, those with doctor-diagnosed stable asthma who sought care at hospital outpatient clinics were recruited. These children with stable asthma had not experienced an exacerbation or increased use of short-acting β_2_-agonists in the 2 weeks before sputum induction. A control group of children who had no history of wheeze or asthma were recruited from surgical wards and general outpatient clinics. All aspects of this study were approved by the East Midlands Research Ethics Committee (reference number 09/H0403/92). Written, informed consent was obtained from the legal guardians of all children prior to enrolment.

### Sputum Collection

#### Children With Acute Asthma

Children with an acute exacerbation of asthma seeking care at the ED were approached to take part in the study. Prior to sputum collection, all children performed either spirometry to measure the FEV_1_ or a peak expiratory flow measurement to ensure study safety. No child with a lung function (either FEV_1_ or peak expiratory flow) < 50% predicted underwent sputum induction, and only spontaneously expectorated sputum was collected from these children. Where possible, children with acute asthma also had their exhaled nitric oxide measured (NIOX MINO; Aerocrine). Sputum collection followed previously reported protocols^26^ whereby sputum was expectorated either spontaneously, following short-acting β_2_-agonist administration (given as part of clinical management), or following nebulization with 0.9% saline for 30 seconds, and, if needed, 1, 2, or 4 minutes. FEV_1_ or peak expiratory flow was measured after every nebulization. A sample was deemed adequate by means of visual assessment when at least 0.5 mL of sputum containing three or more opaque mucocellular clumps at least 1.5 to 3 mm in size was obtained.

#### Children With Stable Asthma and Control Subjects

Sputum was collected from children with stable asthma and control children following nebulization with hypertonic saline (at a concentration of 3% and, if needed, increasing to 4% or 5%) by using a high-flow ultrasonic nebulizer (Omron) as previously described.^27^ Prior to induction with hypertonic saline, spirometry following American Thoracic Society/European Respiratory Society guidelines was performed by each child, including postsalbutamol FEV_1_ that was taken as the baseline FEV_1_.^28^

### Sputum Processing and Mucin Quantification

Within 2 hours of collection, sputum plugs were extracted and analyzed for leukocyte differential cell profile, as described previously^27^; see e-Appendix 1 for detailed methods. The remaining sputum sample was frozen at −80°C until mucin quantification. Once defrosted, each sample was solubilized in 4 × 8 M guanidinium chloride (weight by volume) in a cold room under gentle agitation for 2 to 5 days. Each sample was then dialyzed against 6 M urea, and MUC5AC and MUC5B glycoforms were quantified by means of Western blotting after agarose gel electrophoresis.^20^ Mucin quantification was performed blind to the clinical status of the patient. More details are provided in e-Appendix 1.

### Analysis of Mucin Size Distribution by Means of Rate-Zonal Centrifugation

The size distributions of MUC5B and MUC5AC were investigated in the sputum obtained from control subjects and samples from children with acute asthma and following recovery as previously described by Sheehan and Carlstedt.^29^ Briefly, solubilized gel samples were layered onto preformed 6- to 8-M guanidinium chloride gradients and spun at 40,000 rpm (approximately 210,000 g) for 2.5 hours at 15°C in a rotor (Beckman SW 40 Ti; Beckman Coulter). Tubes were emptied from the top into 12 fractions and analyzed for MUC5AC and MUC5B by means of immunodetection following transfer to nitrocellulose by slot blotting.

### Statistics

The concentrations of MUC5AC and MUC5B and the ratio of MUC5B:MUC5AC were log transformed to achieve a normal distribution. Between-group comparisons (control subjects and those with stable or acute asthma) were performed using the independent *t* test. Geometric means and log SDs are reported. Categorical variables were analyzed with a Fisher exact test. The Spearman rank correlation coefficient was used to investigate the relationship between sputum eosinophils and neutrophils and mucin concentrations. A *P* value < .05 was considered significant. Statistical analysis was performed using SPSS version 22 for Windows (SPSS).

## Results

### Subjects

Thirty-eight children participated in this study. Sputum inductions were well tolerated by all children. Sputum samples suitable for analysis of inflammatory cell count and mucin concentration were obtained from all participants. The demographic data and clinical characteristics of each group are shown in Table 1. There were no differences among the three groups in baseline demographic characteristics; however, the stable asthma group had more severe asthma (evidenced by a higher British Thoracic Society asthma guideline treatment step)^30^ and had had asthma diagnosed for longer than had the acute asthma group. Postbronchodilator FEV_1_ was significantly lower in the acute asthma group than in the stable asthma group and in control subjects.

**Table 1:** Demographic and Asthma Characteristics of Children With Acute Asthma, Children With Stable Asthma, and Control Subjects

Characteristic	Control Subjects (n = 10)	Children With Stable Asthma (n = 15)	Children With Acute Asthma (n = 13)	*P* value
Male patients, No. (%)	6 (60.0)	9 (60.0)	9 (69.2)	ns
Age, median (range), y	12 (7-15)	13 (6-15)	12 (6-15)	ns
Ethnicity, No. (%)				
White	8 (80.0)	11 (73.3)	8 (61.5)	ns
South Asian	0 (0)	1 (6.7)	2 (15.4)	ns
Afro-Caribbean	0 (0)	3 (20.0)	1 (7.7)	ns
Other	2 (20.0)	0 (0)	2 (15.4)	ns
BMI, median (range), kg/m^2^	18.4 (14.7-27.1)	19.3 (15.4-28.3)	17.3 (13.4-22.2)	ns
FEV_1_ % predicted, median (range)	95.4 (79.3-100.8)	89.9 (69.0-124.0)	68.4 (42.9-109.8)	.003^a^
eNO,^b^ median (range), ppb	15 (6-59)	43 (9-169)	15 (0-58)	.025^c^
BTS treatment step, No. (%)				
0	…	0 (0)	1 (7.7)	ns
1	…	2 (13.3)	4 (30.8)	ns
2	…	0 (0)	2 (15.4)	ns
3	…	2 (13.3)	4 (30.8)	ns
4	…	8 (53.3)	1 (7.7)	ns
5	…	3 (20.0)	1 (7.7)	.008^a^
Years with asthma diagnosed, median (range)	…	9 (0-15)	4 (0-14)	.010^a^
Atopic,^d^ No. (%)	1 (10)	12 (80.0)	9 (69.2)	ns
Sputum inflammatory cell profile, median (range)				
Total leukocyte count ×10^6^/mL	0.13 (0.05-3.77)	0.22 (0.04-3.36)	1.16 (0.02-3.26)	ns
Sputum neutrophils ×10^6^/mL	0.04 (0.02-0.88)	0.05 (0.01-2.94)	0.32 (0.02-2.67)	.018^a^
% neutrophils of total leukocytes	44.5 (8.0-96.75)	25.5 (8.5-91.25)	79.3 (7.75-96.5)	.032^a^
Sputum eosinophils ×10^6^/mL	0.00 (0.0-0.00)	0.01 (0.0-0.60)	0.08 (0.0-1.17)	.001^c^
% eosinophils of total leukocytes	0.13 (0.0-2.0)	5.0 (0.0-56.0)	5.0 (0.0-68.50)	.008^c^
Sputum macrophages ×10^6^/mL	0.07 (0.0-2.71)	0.12 (0.0-0.78)	0.21 (0.0-0.85)	ns
% macrophages of total leukocytes	52.4 (1.75-88.8)	51.0 (5.25-86.5)	12.0 (2.50-68.0)	.007^a^

BTS = British Thoracic Society; eNO = exhaled nitric oxide; ns = not significant; ppb = parts per billion.

a *P* value between those with acute asthma vs those with stable asthma.

b eNO was performed in nine children with acute asthma owing to poor technique in three children and unavailability of NIOX MINO (Aerocrine) in one child.

c *P* value between those with stable asthma vs control subjects.

d Atopic was classed as current history of either hay fever or eczema.

### Concentration of MUC5AC and MUC5B in the Sputum of Control Subjects and Children With Stable or Acute Asthma

**Figure 1:**
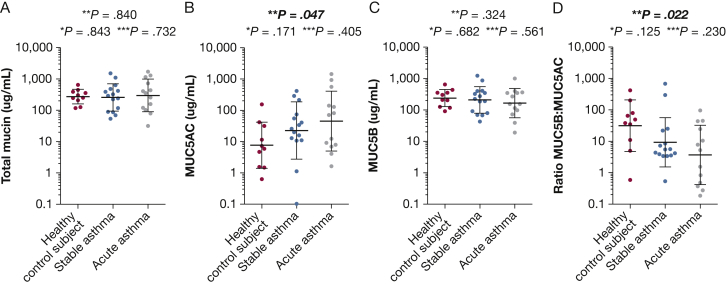
Mucin quantification by means of Western blotting after agarose electrophoresis in sputum from children with acute asthma, children with stable asthma, and control subjects. A, Total mucin. B, MUC5AC. C, MUC5B. D, MUC5B:MUC5AC ratio. • = an individual recruit. The horizontal black lines represent the geometric mean with SD. P values were calculated using the student *t test*; bold and italicized indicates statistically significant P values. * = P value between control subjects and those with stable asthma. ** = P value between control subjects and those with acute asthma. *** = P value between those with acute asthma and those with stable asthma.

Concentration of MUC5AC showed considerable variation among the three groups studied, with geometric means of 7.6, 22.4, and 44.7 μg/mL in the control subject, stable asthma, and acute asthma groups, respectively (Fig 1). MUC5AC differed significantly in concentration between those with acute asthma and control subjects (*P* = .047), with a mean difference of 0.17 (95% CI, 0.03-0.97). Concentrations of MUC5B were similar in all three groups, with geometric means of 238.5, 208.4 and 165.9 μg/mL for the control subject, stable asthma, and acute asthma groups, respectively (Fig 1). The MUC5B:MUC5AC ratios between control subjects and those with acute asthma were significantly decreased (geometric means of 31.6 vs 3.71; *P* = .022), with a mean difference of 8.49 (95% CI, 1.4-51.1). In control subjects and children with stable asthma, the geometric means of the MUC5B:MUC5AC ratios were 31.6 and 9.33, respectively (*P* = .119), and the mean difference was 3.37 (95% CI, 0.71-15.92).

### MUC5B Glycoforms

**Figure 2:**
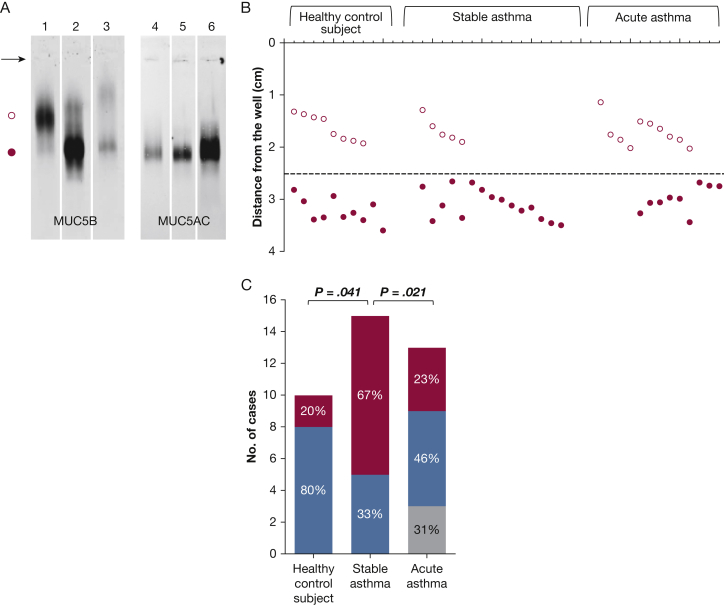
Detection of MUC5AC and MUC5B glycoforms by using Western blotting. A, Western blots of MUC5B and MUC5AC. Western blots of reduced sputum after agarose electrophoresis; MUC5B (lanes 1-3) and MUC5AC (lanes 4-6) were detected with MAN5BI and MAN5ACI polyclonal antibodies, respectively. The arrow shows the position of the wells. ○ = the low-charge glycoform of MUC5B; ● = the high-charge glycoform of MUC5B. The data shown are to exemplify the variation in migration between glycoforms and not to represent samples specifically from each study group. B, Designation of MUC5B glycoforms by means of eletrophoretic mobility. The distance of migration of the MUC5B bands detected in each sample was measured. MUC5B was designated as low-charge glycoform if migration was < 2.5 cm (open circles above the line) and high-charge glycoforms if migration was > 2.5 cm (filled circles below the line). C, Distribution of MUC5B glycoforms. Bar graph of the number of individuals with both glycoforms (blue), high-charge-only glycoform (red), or low-charge-only glycoform (gray). The percentages of MUC5B glycoform categories found within each group (control subject, stable asthma, and acute asthma groups) are shown.

We found significant differences in MUC5B glycoforms between the groups (Fig 2). The sputum of most control children contained a mixture of both the high- and low-charge MUC5B glycoforms. In contrast, the high-charge MUC5B glycoform alone was present in approximately two-thirds (67%) of children with stable asthma, with the remaining 33% consisting of both high- and low-charge glycoforms. This finding was significantly different from that in control subjects in whom 80% had both high- and low-charge glycoforms and 20% had the high-charge-only glycoform (*P* = .041, Fisher exact test). The low-charge MUC5B glycoform alone was present only in the sputum from children with acute asthma, which occurred in approximately one-third (31%) of children.

### Validation of Mucin Quantification and Evaluation of Salivary Proteins in Sputum Samples by Means of Mass Spectrometry

We validated Western blot mucin quantification by using tandem mass spectrometry to assess for mucin proteolysis, which could lead to underestimation of the reported mucin concentration as reported in cystic fibrosis.^31^, ^32^ Tryptic mapping of MUC5B and MUC5AC showed that peptides were distributed throughout the mucin polypeptides in sputum from all groups, suggesting proteolysis was unlikely (e-Figs 1, 2). This validation enabled us to assess for salivary contamination also. However, no differences in salivary proteins between the different sample groups were observed (e-Fig 3). See e-Appendix 1 also for detailed methods.

### Mucin Size Distribution During Acute Asthma and Following Recovery

**Figure 3:**
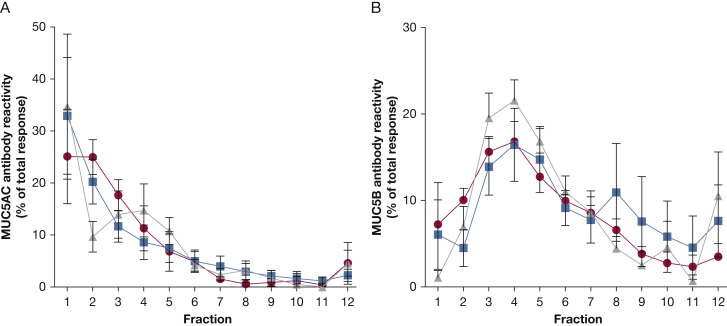
Analysis of mucin size distribution by means of rate-zonal centrifugation. The sample was layered onto preformed 6- to 8-M guanidinium chloride gradients and centrifuged for 2.5 hours at 40,000 rpm and 15°C. Fractions (1 mL) were unloaded from the top of the tube and immunoblotted for MUC5AC and MUC5B by using mucin-specific antisera. The average size distributions for MUC5AC (A) and MUC5B (B) are shown. Each value represents the mean, with SD error bars. ● = Control subjects (n = 4); ▪ = children with asthma acute exacerbation (n = 7); and ▲ = children with acute asthma at recovery. Fraction 1 = lowest density; fraction 12 = highest density.

Rate-zonal centrifugation to compare the size distribution of MUC5AC and MUC5B was performed on sputum samples from a separate group of seven children (five male; median age, 11 years; age range, 7-16 years) in whom acute exacerbation and recovery sputum were available and four control children (two male; median age, 12.5 years; age range, 5-16 years). The data showed that the samples all contained mucin polymers of varying size, and no discernible difference in MUC5AC and MUC5B size distribution was observed when we compared control subject, acute asthma group, and recovery sputum (Fig 3). This finding suggests that the size distribution of MUC5AC and MUC5B in all samples was similar and that mucin composition, rather than mucin size distribution, likely contributes to airway mucus obstruction in pediatric acute asthma.

### Relationship Between Airway Inflammation and MUC5AC and MUC5B in Those With Stable or Acute Asthma

Previous studies reported an association between sputum eosinophils, airway mucins,^9^ and MUC5AC.^13^ The proportion of sputum eosinophils was significantly greater in children with asthma than in control subjects (Table 2), and we found that total mucin, MUC5AC, and MUC5B were correlated with sputum eosinophils in children with stable disease. We also found that in children with stable or acute asthma, the MUC5AC sputum concentration was significantly correlated with the number of sputum neutrophils (Table 2).

**Table 2:** Correlation Between Numbers of Sputum Inflammatory Cells and Total Mucin, MUC5AC, and MUC5B

Eosinophils or Neutrophils	Control Subjects, μg/mL (n = 10)			Children With Stable Asthma, μg/mL (n = 15)			Children With Acute Asthma, μg/mL (n = 13)		
	Total Mucin	MUC5AC	MUC5B	Total Mucin	MUC5AC	MUC5B	Total Mucin	MUC5AC	MUC5B
Sputum eosinophils ×10^6^/mL, *r* (*P* value)	…^a^	…^a^	…^a^	**0.635 (.011)**	**0.566 (.028)**	**0.562 (.029)**	−0.110 (.720)	−0.055 (.858)	−0.287 (.343)
Sputum neutrophils ×10^6^/mL, *r* (*P* value)	−0.013 (.973)	0.075 (.836)	−0.031 (.931)	0.467 (.079)	**0.553 (.032)**	0.326 (.236)	**0.363 (.223)**	0.670 (.012)	−0.143 (.642)

Bold *P* values indicate *P* < .05. *r* = Spearman rank correlation coefficient.

a No data because of very low levels of eosinophils in sputum from control subjects.

## Discussion

To our knowledge, this is the first study to characterize airway mucin composition in children with asthma. We found that MUC5AC concentration was increased in children with asthma compared with that in control subjects, particularly in children with acute asthma, resulting in a significant change in the MUC5B:MUC5AC ratio. We also found significant differences in the sputum composition of MUC5B glycoforms between the sample groups. In most control subject samples, MUC5B was present as a mixture of the high- and low-charge glycoforms; a small number of control subject samples contained high-charge MUC5B only. In contrast, two-thirds of samples from patients with stable asthma consisted of the high-charge glycoform of MUC5B alone. Strikingly, in one-third of acute asthma sputum, only the low-charge MUC5B glycoform was present.

MUC5B glycoforms are reported rarely because they are not easily detected using messenger RNA or enzyme-linked immunosorbent assay methods. Moreover, lectin or carbohydrate-specific antibodies are not necessarily specific for a particular mucin gene product or its glycoforms. The majority of published studies report total mucin concentrations or MUC5AC alone.^9^, ^21^, ^33^, ^34^ A recent study in adults using a novel enzyme-linked immunosorbent assay method to differentiate MUC5B and MUC5AC reported similar findings to ours, with a relative decrease in MUC5B compared with MUC5AC in acute asthma; however, this technique did not differentiate between MUC5B glycoforms^13^ MUC5B has been detected in sputum plugs obtained after fatal asthma, although MUC5B glycoforms were not distinguished.^12^, ^14^ However, high concentrations of the low-charge MUC5B glycoform was present in mucus plugs obtained post mortem from an adult who died in status asthmaticus.^11^ Why different MUC5B glycoforms are secreted into the airway is not well understood, and their impact on mucus function requires clarification.

Recent findings by Bonser et al^14^ have demonstrated that altered mucin composition (increased MUC5AC, reduced MUC5B) dramatically impairs mucus transport in asthma, likely contributing to the viscous plugs seen in acute disease. In our study population, lung function was significantly reduced in those with acute asthma compared with that in those with stable asthma. The alterations in mucin composition we observed, namely increased MUC5AC and low-charge MUC5B, may contribute to a pathologic mucus gel with altered mucus biophysical properties leading to airway obstruction.

Asthma-associated airway inflammatory cells and their mediator products have been shown to induce airway mucin synthesis and secretion,^5^, ^9^, ^35^, ^36^, ^37^ and this is likely affected by whether the patient is in an acute exacerbation or more stable state of disease. Jinnai et al^9^ reported an association between airway eosinophils and total mucin concentration in the sputum of adults with stable asthma; they did not distinguish between MUC5B or MUC5AC. We found a similar relationship between eosinophils and concentrations of MUC5B and MUC5AC in stable asthma. In both stable and acute asthma, MUC5AC was significantly correlated with airway neutrophils only. Acute exacerbations of asthma are associated with neutrophilic airway inflammation.^38^ Neutrophil elastase can break down mucins, and this degradation can be inhibited by proteins present in the airway during acute asthma.^15^ Inhibiting mucin degradation may impair mucociliary clearance leading to airway mucus obstruction and contributing to the reduced lung function we observed in acute disease.

Studies of this nature are complex and constitute a not insignificant research burden, especially in children. Such considerations limit the number of tests, including repeat sampling and bronchoscopic studies, allowed by research ethics committees. Further studies would benefit from more information regarding viral exposure during acute asthma exacerbation and from measuring the concentration of MUC5AC and MUC5B during an acute asthma exacerbation and at recovery in the same patient. Although ethical considerations prohibited the use of hypertonic saline in those with acute asthma, it is unlikely that differences in the method of obtaining sputum would have confounded our results. For example, we found no association between concentration of MUC5AC or MUC5B and its glycoforms and whether the sputum sample had been obtained by means of spontaneous expectoration or hypertonic saline. This finding is in keeping with those of previous reports in which the investigators compared levels of MUC5AC and MUC5B in sputum obtained by means of nebulization with differing strengths of saline or that obtained by means of spontaneous expectoration^19^ MUC2 was not measured because quantitative immunoblotting and mass spectrometry analysis of sputum from healthy subjects and individuals with asthma, cystic fibrosis, or COPD showed little evidence for the presence of MUC2.^39^

## Conclusions

We found important differences in sputum mucin composition between healthy children and children with asthma. In children with asthma, we observed an increased MUC5AC concentration, particularly in those with acute disease. A greater prevalence of low-charge MUC5B was also observed in samples from those with acute asthma. We found no differences in airway mucin size distribution between those with acute asthma and those with stable asthma, suggesting that it is the altered mucin composition that contributes to airway obstruction in acute asthma and that strategies targeting MUC5AC and low-charge MUC5B may unplug the airways.

## Supplementary Data

e-Online Data
