# Supplementary material for: MUC5AC and a Glycosylated Variant of MUC5B Alter Mucin Composition in Children With Acute Asthma
Source: Chest. 2017 Jul 14;152(4):771–9. doi: 10.1016/j.chest.2017.07.001 (PMC5624091; doi:10.1016/j.chest.2017.07.001)
Supplement: e-Online Data [file mmc1.pdf]

CHEST Online Supplement

MUC5AC and a Glycosylated Variant of MUC5B Alter Mucin Composition in Children With Acute Asthma

Kathryn G. Welsh, MBChB; Karine Rousseau, PhD; Gemma Fisher, MBChB; Luke R. Bonser, PhD; Peter Bradding, DM; Chris E. Brightling, PhD; David J. Thornton, PhD; and Erol A. Gaillard, PhD

CHEST 2017; 152(4):771-779

Online supplements are not copyedited prior to posting and the author(s) take full responsibility for the accuracy of all data.

© 2017 AMERICAN COLLEGE OF CHEST PHYSICIANS. Reproduction of this article is prohibited without written permission from the American College of Chest Physicians. See online for more details. **DOI:** 10.1016/j.chest.2017.07.001

e-Appendix 1.

Methods

Leucocyte differential cell profile

All samples underwent processing to obtain a leucocyte differential cell profile within two hours of sputum collection. The method for obtaining the leucocyte differential cell count has been described elsewhere [1]. Briefly, sputum plugs were removed from the sample using blunt forceps and diluted 4 x (w/v) with 0.1% dithiothreitol (DTT). The remaining sputum sample was frozen at -80°C. Following reduction with DTT, the samples were filtered, centrifuged and the pellet re-suspended in D-PBS to a concentration of 0.5x10^{6} cells/ml (following total cell count and assessment of cell viability by trypan blue exclusion method). A cytospin slide of cell suspension was stained using the DiffQuik method (Dade Behring, Dudingen, Switzerland) and a differential leucocyte count was performed by counting 400 non-squamous cells. Leucocyte differential cell counts were performed by a highly experienced individual, who was blinded to the clinical patient data. Total cell counts (x10^{6}/mL) and differential inflammatory cell counts are reported.

Mucin Quantification by Western blotting

After solubilization of sputum with 4M guanidinium chloride (GdmCl) each sample was dialyzed against 6M urea. Different volumes of dialyzed samples were blotted onto a nitrocellulose membrane (20µl, 10µl and 5µl for MUC5B and 10µl, 5µl and 2.5µl for MUC5AC) and their MUC5B and MUC5AC content estimated by dot immunoblotting with the mucin-specific antisera MAN-5BI and MAN-5ACI [2–4] . The results from immunoblotting were used to estimate at which dilution each sample should be used for mucin quantification by Western blotting after agarose gel electrophoresis.

In brief, 0.7% agarose gels were prepared in TAE buffer (40mM Tris acetate, 1mM EDTA, pH 8.0 containing 0.1% (w/v) SDS) [5]. Several dilutions of mucin standards were loaded twice onto each gel (0.52ng, 0.325ng, 0.26ng and 0.1625ng of MUC5AC and 1.571µg, 0.942µg, 0.785µg, 0.471µg and 0.393µg of MUC5B) in addition to 4 different dilutions of each sample. The samples were reduced by adding 10mM DTT to the loading buffer (1M Tris acetate, 50mM EDTA, 8M urea, 1% (w/v) SDS, 40% glycerol and Bromophenol blue) followed by heating at 100°C for 5 minutes prior to loading and electrophoresed for 3 hours at 60 volts. The glycoproteins were then transferred onto nitrocellulose membrane (0.42µm mesh size) for 1 hour and 30 minutes by vacuum transfer (50 mBar). MUC5B and MUC5AC were detected using the MAN-5BI and MAN-5ACI polyclonal antisera respectively. A goat anti-rabbit Infra-Red Dye 800CW secondary antibody (Li-Cor, Lincoln, Nebraska, USA) followed by analysis with the Odyssey densitometer was used to visualize the mucins. The intensity of each band was determined with the Odyssey 2.1 software. For each gel, a standard curve of the mucin standards (see below for details of the preparation of mucin standard

Online supplements are not copyedited prior to posting and the author(s) take full responsibility for the accuracy of all data.

solutions) was calculated; an R^2 above 0.95 was considered acceptable. For each sample, densitometry values were re-adjusted for dilution and the standard deviation calculated; the samples were considered quantifiable only if the percentage of deviation was less than 15% of the average of the dilutions. A mean based on a minimum of 3 values was used for each sample.

Purified mucin solutions at known concentrations were used as standards for mucin quantitation. MUC5B was purified from saliva while MUC5AC was isolated from HT29-A1 cell culture media [2]. Prior to purification, the HT29-A1 cell culture media was 20 times concentrated using a tangential flow concentrator fitted with a 300KDa cassette (from PALL Corporation); this was not necessary for saliva. Concentrated cell culture media and saliva were then dissolved 1:1 in 8M GdmCl and mucins were purified from other proteins and nucleic acids by two consecutive cesium chloride density gradient centrifugations as described previously [4–6]. The first density gradient centrifugation was performed at a starting density of 1.4g/ml in 4M GdmCl to remove proteins and the second at a starting density of 1.5g/ml in 0.2M GdmCl to remove nucleic acids. Each gradient was unloaded into 20 fractions and each was tested for mucins content by periodic acid-Schiff (PAS) and antibody stain on slot blots as described previously [7]. Following the second density gradient centrifugation the mucin containing fractions were pooled and concentrated using a Vivaspin 20 with a molecular weight cut off of 100kDa. An aliquot of the concentrated purified mucins was then reduced and carboxymethylated and analyzed by refractometry to determine their concentration. Briefly, purified mucins were chromatographed on a Superose 6 column in 0.2M NaCl/1mM EDTA followed by detection with an in-line Optilab rEX refractometer (Wyatt Technology Corporation) The analyses were performed using a refractive index increment (*dn/dc*) of 0.165 [8] and yielded a concentration of 10.4 mg/ml for MUC5AC and 23.55 mg/ml for MUC5B.

Mass spectrometry to validate mucin quantification by Western blotting and to evaluate salivary proteins in sputum samples

Nine sputum samples (3 acute asthma, 3 stable asthma and 3 healthy control) were reduced with 10mM DTT in Tris-HCl pH8 and carboxymethylated with 25mM iodoacetamide in the same buffer. Vivaspin 6 spin filters with a MWCO of 5000Da were used to concentrate the samples down to 100µl and buffer exchange into 50mM ammonium bicarbonate containing 2M urea. The samples were then digested with trypsin (5µg). The resulting peptides were collected by centrifugation and desalted using a C18 Zip Tip. Peptides were separated by liquid chromatography and analysed inline by tandem mass spectrometry (LC-MS/MS) using an UltiMate® 3000 Rapid Separation LC (RSLC, Dionex Corporation, Sunnyvale, CA) coupled to a LTQ Velos Pro (Thermo Fisher Scientific, Waltham, MA) mass spectrometer. Peptide mixtures were separated using a gradient from 92% A (0.1% formic acid in water) and 8% B (0.1% formic acid in acetonitrile) to 33% B, in 44min at 300nL min-1, using a 250mm x 75µm i.d. 1.7mM BEH C18 analytical column (Waters). Peptides

Online supplements are not copyedited prior to posting and the author(s) take full responsibility for the accuracy of all data.

were selected for fragmentation automatically by data dependant analysis. Data produced were searched using Mascot (Matrix Science UK), against the UniProt database. Data were analysed using Progenesis QI for Proteomics (Waters).

We assessed potential proteolysis of the mucins by analyzing the peak intensities for peptides in the N-terminal domain (NTD), the internal Cys-domains (CD) and the C-terminal domain (CTD). If proteolysis had occurred one might expect this ratio to change between the sample groups. Examining the ratio of intensities for NTD:CD and CTD:CD for each sample showed no marked change in ratios between the different sample groups. These data indicate that the immune-reactivity of MUC5B and MUC5AC is not affected by proteolysis in the samples analysed in this study (Supplementary data Figure E1 and E2). It is noteworthy that MUC2 peptides were not identified in the peptide mapping experiments, suggesting that MUC2, if present, is a very minor component in our samples.

We evaluated the presence of salivary proteins in sputum samples by using the MS/MS data. Twenty-three different salivary proteins were identified and the mean number of spectral counts for these proteins [9–12] showed no major difference in salivary proteins between each group (Supplementary data Figure E3). We therefore conclude that salivary contamination is unlikely to have affected the results of mucin concentrations in our samples.

Online supplements are not copyedited prior to posting and the author(s) take full responsibility for the accuracy of all data.

References

1. Pizzichini E, Pizzichini MMM, Efthimiadis A.; Evans S, Morris MM, Squillace, D, Gleich, GJ, Dolovich, J, Hargreave F. Indices of airway inflammation in induced sputum: reproducibility and validity of cell and fluid phase measurements. *Am J Respir Crit Care Med* 1996;154:308–17.
2. Kirkham S, Sheehan JK, Knight D, Richardson PS, Thornton DJ. Heterogeneity of airways mucus: variations in the amounts and glycoforms of the major oligomeric mucins MUC5AC and MUC5B. *Biochem J* 2002;361(3):537–46.
3. Thornton DJ, Carlstedt I, Howard M, Devine PL, Price MR, Sheehan JK. Respiratory mucins: identification of core proteins and glycoforms. *Biochem J* 1996;316(3):967–75.
4. Thornton DJ, Howard M, Khan N, Sheehan JK. Identification of two glycoforms of the MUC5B mucin in human respiratory mucus. Evidence for a cysteine-rich sequence repeated within the molecule. *J Biol Chem* 1997;272(14):9561–6.
5. Thornton DJ, Howard M, Devine PL, Sheehan JK. Methods for separation and deglycosylation of mucin subunits. *Anal Biochem* 1995;227(1):162–7.
6. Thornton DJ, Khan N, Mehrotra R, et al. Salivary mucin MG1 is comprised almost entirely of different glycosylated forms of the MUC5B gene product. *Glycobiology* 1999;9(3):293–302.
7. Thornton DJ, Carlstedt I, Sheehan JK. Identification of glycoproteins on nitrocellulose membranes and gels. *Mol Biotechnol* 1996;5(2):171–6.
8. Sheehan JK, Brazeau C, Kutay S, et al. Physical characterization of the MUC5AC mucin: a highly oligomeric glycoprotein whether isolated from cell culture or in vivo from respiratory mucous secretions. *Biochem J* 2000;347(1):37–44.
9. Castagnola M, Inzitari R, Fanali C, et al. The surprising composition of the salivary proteome of preterm human newborn. *Mol Cell Proteomics* 2011;10(1):M110.003467.
10. Sousa-Pereira P de, Cova M, Abrantes J, et al. Cross-species comparison of mammalian saliva using an LC-MALDI based proteomic approach. *Proteomics* 2015;15(9):1598–607.
11. Sivadasan P, Gupta MK, Sathe GJ, et al. Human salivary proteome--a resource of potential biomarkers for oral cancer. *J Proteomics* 2015;127:89–95.
12. Kesimer M, Kirkham S, Pickles RJ, et al. Tracheobronchial air-liquid interface cell culture: a model for innate mucosal defense of the upper airways? *Am J Physiol Lung Cell Mol Physiol* 2009;296(1):L92–L100.

Online supplements are not copyedited prior to posting and the author(s) take full responsibility for the accuracy of all data.

e-Figure 1: MUC5B mass spectrometry peptide coverage

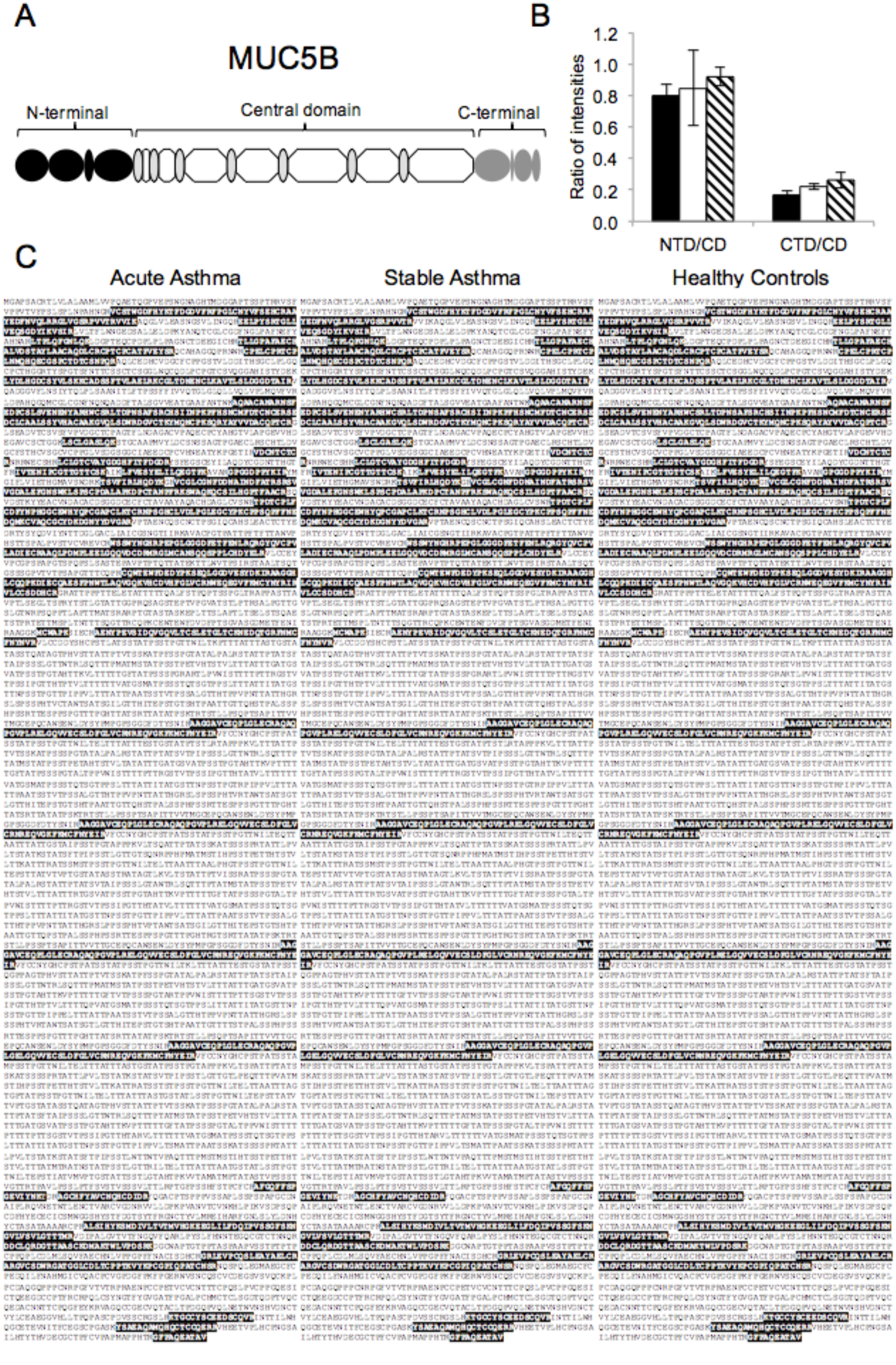

Online supplements are not copyedited prior to posting and the author(s) take full responsibility for the accuracy of all data.

e-Figure 1: A. Schematic representation of MUC5B polypeptide. The N-terminal (black), C-terminal (dark grey) domains and cysteine-rich regions of the central domain (light grey) are the regions of MUC5B from which tryptic peptides can be derived for mass spectrometry analysis. **B.** Ratio of the total intensity of the peptides detected in the different domains of MUC5B for acute samples (black), stable samples (white) and healthy controls (stripes). **C.** Peptides identified by mass spectrometry in sputum from 3 individuals with acute asthma, 3 individuals with stable asthma and 3 healthy individuals that matched the human MUC5B sequence (accession number Q9HC84) are highlighted in the black boxes.

Online supplements are not copyedited prior to posting and the author(s) take full responsibility for the accuracy of all data.

e-Figure 2: MUC5AC mass spectrometry peptide

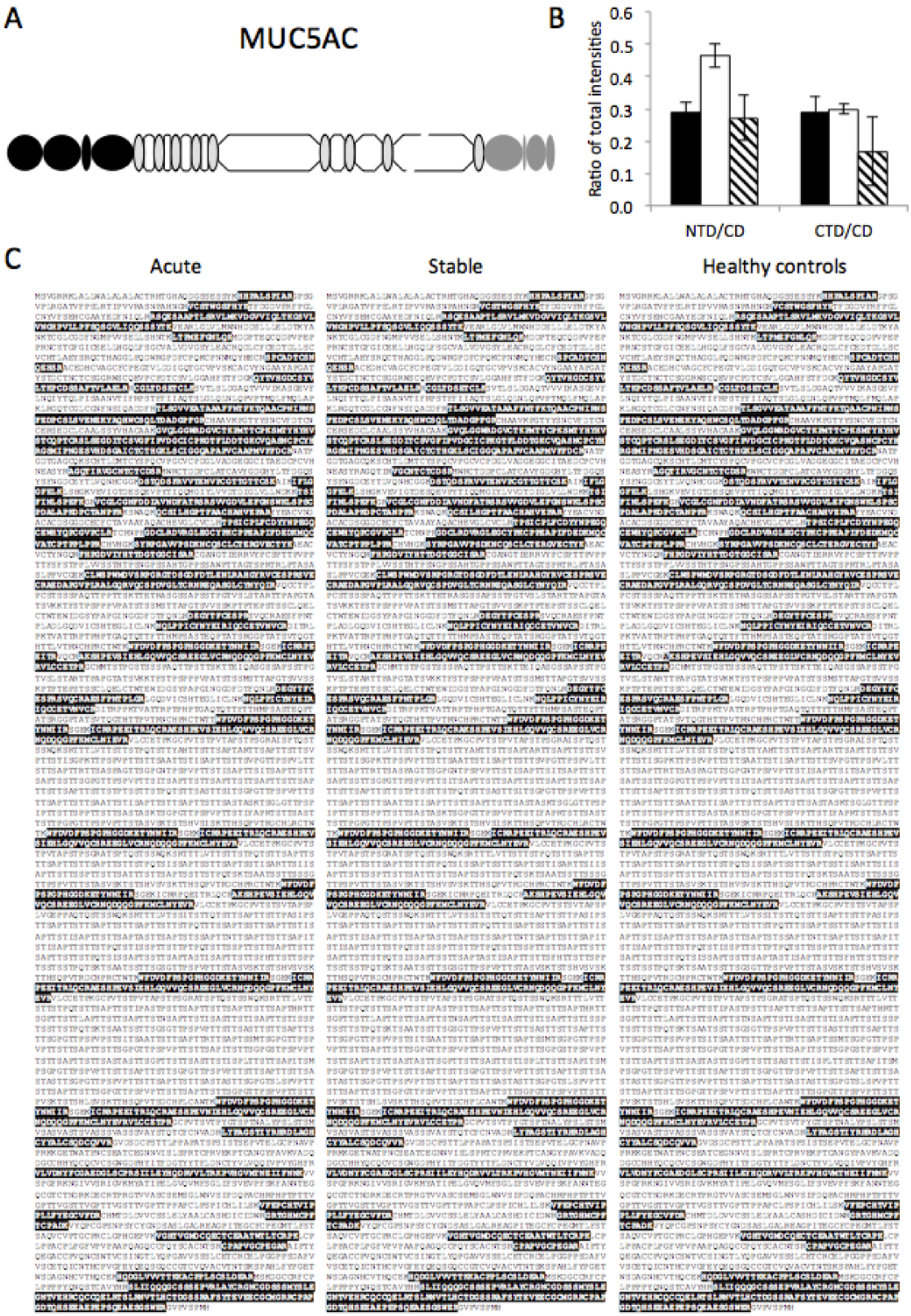

e-Figure 2: A. Schematic representation of MUC5AC polypeptide. The N-terminal (black), C-terminal (dark grey) domains and cysteine-rich regions of the central domain (light grey) are the regions of MUC5AC from which tryptic peptides can be derived for mass spectrometry analysis. **B.** Ratio of the total intensity of the peptides detected in the different domains of MUC5AC for acute samples (black), stable samples (white) and healthy controls (stripes). **C.** Peptides identified by mass spectrometry in the sputum from 3 individuals with acute asthma, 3 individuals with stable asthma and 3 healthy individuals that matched the human MUC5AC sequence (accession number P98088) are highlighted in the black boxes.

Online supplements are not copyedited prior to posting and the author(s) take full responsibility for the accuracy of all data.

e-Figure 3: Mean spectral counts for the major salivary proteins detected by tandem mass spectrometry in sputum from acute asthma, stable asthma and healthy controls

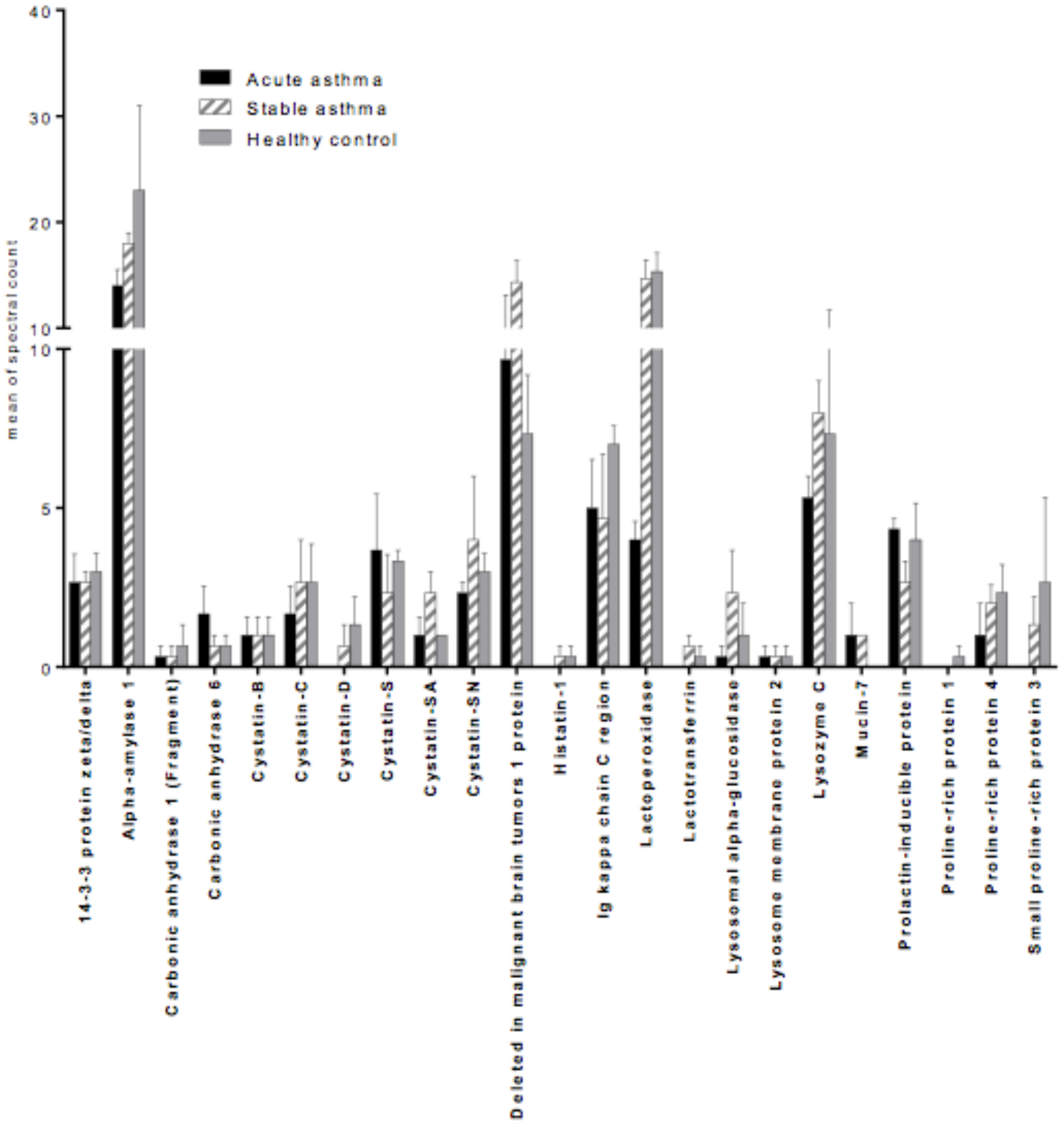

e-Figure 3: 3 samples per group were tested. Error bars represent the standard error of the mean.

170229

Online supplements are not copyedited prior to posting and the author(s) take full responsibility for the accuracy of all data.
